# Open Maximal Mucosa-Sparing Functional Total Laryngectomy

**DOI:** 10.3389/fsurg.2017.00060

**Published:** 2017-10-12

**Authors:** Pavel Dulguerov, Naif H. Alotaibi, Stephanie Lambert, Nicolas Dulguerov, Minerva Becker

**Affiliations:** ^1^Department of Oto-Rhino-Laryngology – Head and Neck Surgery, Geneva University Hospitals (HUG), Geneva, Switzerland; ^2^Department of Imaging and Medical Information Sciences, Geneva University Hospitals (HUG), Geneva, Switzerland

**Keywords:** total laryngectomy, aspiration, swallowing, surgical technique, robotics, complications, flap reconstruction

## Abstract

**Background:**

Total laryngectomy after (chemo)radiotherapy is associated with a high incidence of fistula and therefore flaps are advocated. The description of a transoral robotic total laryngectomy prompted us to develop similar minimally invasive open approaches for functional total laryngectomy.

**Methods:**

A retrospective study of consecutive unselected patients with a dysfunctional larynx after (chemo)radiation that underwent open maximal mucosal-sparing functional total laryngectomy (MMSTL) between 2014 and 2016 is presented. The surgical technique is described, and the complications and functional outcome are reviewed.

**Results:**

The cohorts included 10 patients who underwent open MMSTL. No pedicled flap was used. Only one postoperative fistula was noted (10%). All patients resumed oral diet and experienced a functional tracheo-esophageal voice.

**Conclusion:**

MMSTL could be used to perform functional total laryngectomy without a robot and with minimal incidence of complications.

## Introduction

In a landmark article, Lawson et al. (1) described in 2013 a new surgical technique for total laryngectomy: the transoral robotic laryngectomy. The technique involved preparing the supraglottic larynx transorally with pre-epiglottic and retroarytenoid mucosal incisions followed by a progressive dissection around the laryngeal cartilages in an inferior direction. A cervical incision is necessary for the creation of the tracheostomy but the larynx is delivered transorally. The exact amount of transoral vs. transcervical dissection was not specified, but heavy emphasis is placed on the use of endoscopic robotic dissection.

This article is essentially a technical note without clinical details such as the exact indications for the procedure, the duration of the procedure, the number of patients, or the complications associated with procedure.

This article inspired us to modify traditional laryngectomy techniques in cases of a dysfunctional larynx after (chemo)radiation, the so-called functional laryngectomy. The technique retains the main advantages of the transoral robotic laryngectomy: (1) minimal neck incisions, (2) maximal mucosa sparing, (3) minimal pharyngotomy defect, (4) minimal lateral dissection toward the carotid sheath, (5) horizontal closure, and (6) preservation of prelaryngeal muscles, allowing minimizing the risk of fistula.

## Materials and Methods

### Surgical Technique

**Figure 1:**
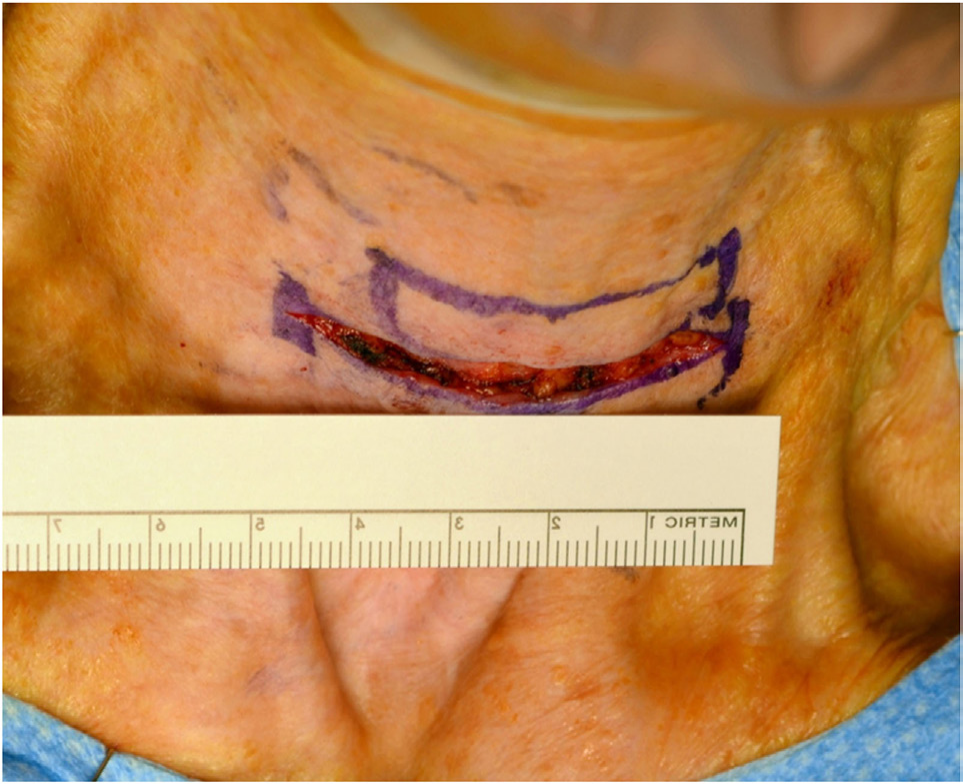
Neck incision for maximal mucosa sparing functional total laryngectomy.

The procedure is performed through a 5- to 6-cm horizontal neck incision, usually by 2-cm lateral extensions of the tracheostomy incision (Figure 1). Since no tracheal sacrifice is necessary, the tracheostomy is planed as high as the first or second tracheal ring. If a previous tracheostomy is placed it is modified as necessary to create an adequate stoma.

**Figure 2:**
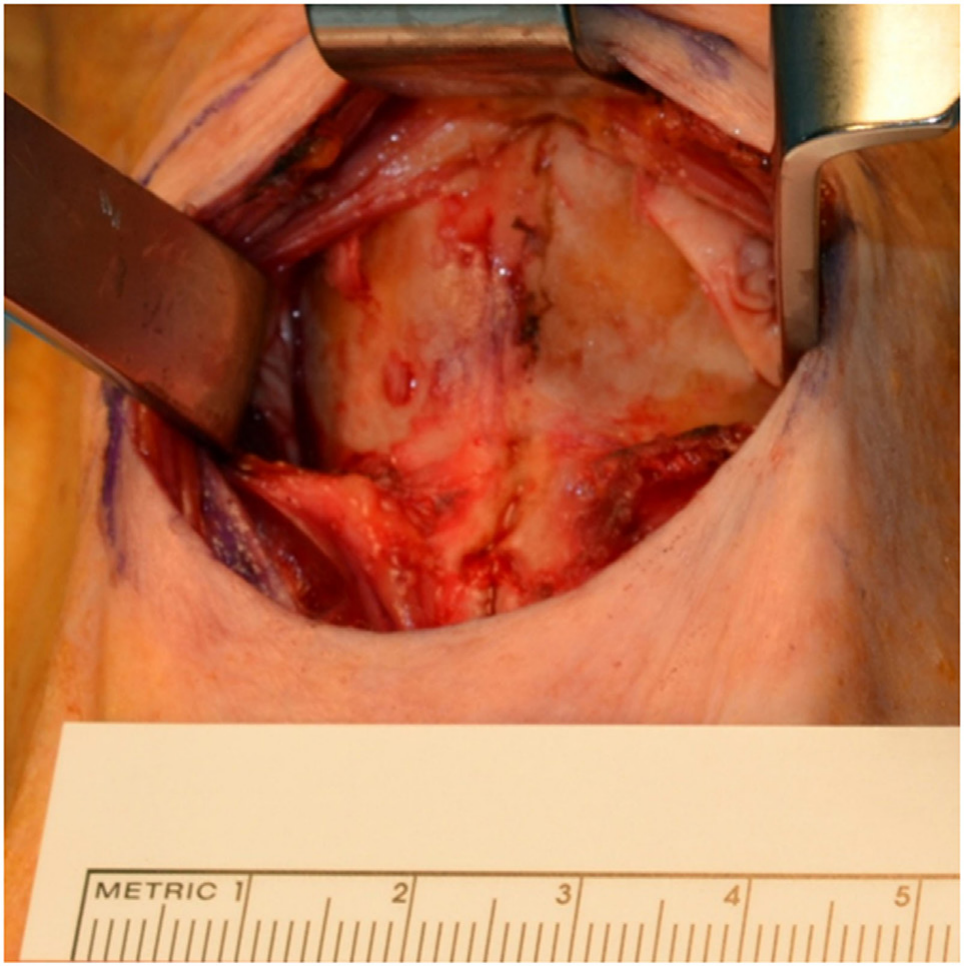
Dissection of anterior aspect of thyroid and cricoid cartilages.

The prelaryngeal musculature is divided in the midline from the hyoid bone to tracheostomy site and the anterior aspect the thyroid and cricoid cartilage dissected. The dissection at the posterolateral aspect of thyroid cartilage is minimal at this point (Figure 2). The attachments of the thyrohyoid muscle are severed at the upper border of the thyroid cartilage. If the upper cornu of thyroid cartilage is prominent it can be sectioned along the natural line of the upper border of the thyroid cartilage and left in place. The tracheostomy is created.

**Figure 3:**
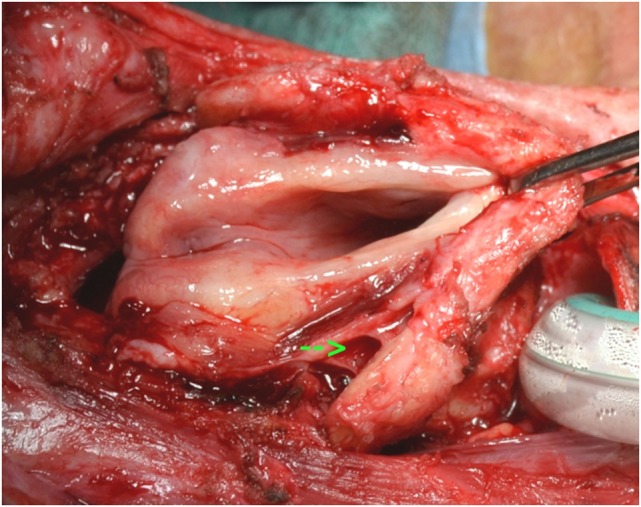
Exposure of larynx in a case after horizontal laryngectomy. Note the freeing of the pyriform sinus on the right (arrow).

After dissection in the pre-epiglottic fat, the epiglottis is felt, grasped, and pulled in an anterior direction. Mucosal incisions are performed following the epiglottic edge, 1–2 mm on the laryngeal side of epiglottis. The larynx in brought in full view by an anterior traction (Figure 3).

**Figure 4:**
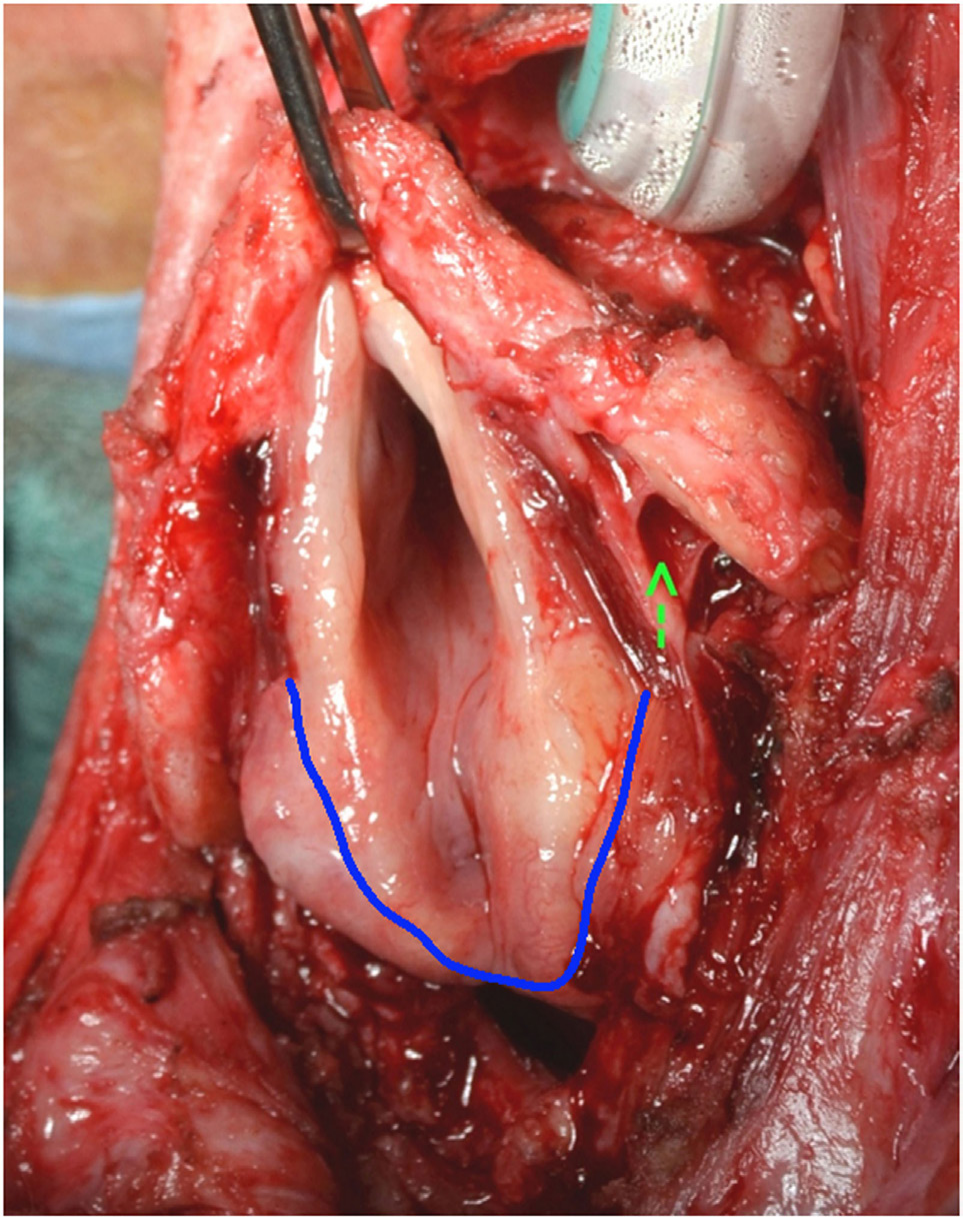
Retroarytenoid incision (blue line).

An incision of the mucosa on the upper border of the posterior cricoid ring, just behind the arytenoids is performed (Figure 4). The dissection proceeds inferiorly, against the posterior aspect of the cricoid cartilage, freeing retrocricoid and esophageal mucosa from the cricoid cartilage and posterior tracheal wall. At some point, the dissection is taken laterally through the paraglottic space to the inner aspect of the thyroid cartilage, the mucosa of the pyriform sinuses is dissected from the thyroid cartilage from the inside out, and the attachments of the constrictor muscles to the lateral border of the thyroid cartilage severed.

**Figure 5:**
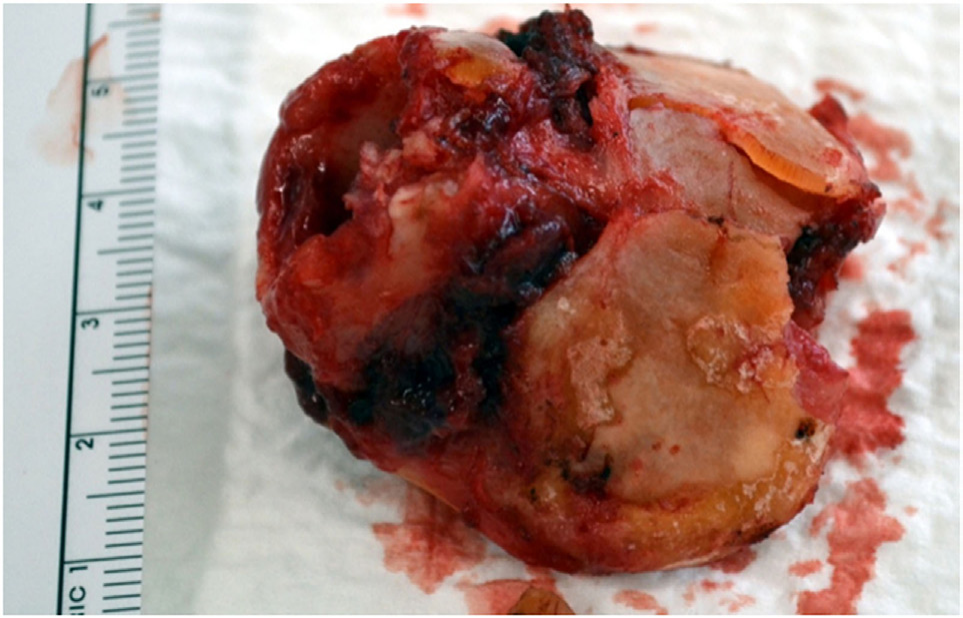
Laryngectomy specimen, essentially made of laryngeal cartilages.

The esophageal musculature is minimally disturbed since the dissection proceeds along the cricoid cartilage and posterior tracheal wall under direct vision. The larynx is than delivered and is basically made up of skeletonized laryngeal cartilages, leaving a pharyngeal defect of about 4–5 cm (Figure 5).

**Figure 6:**
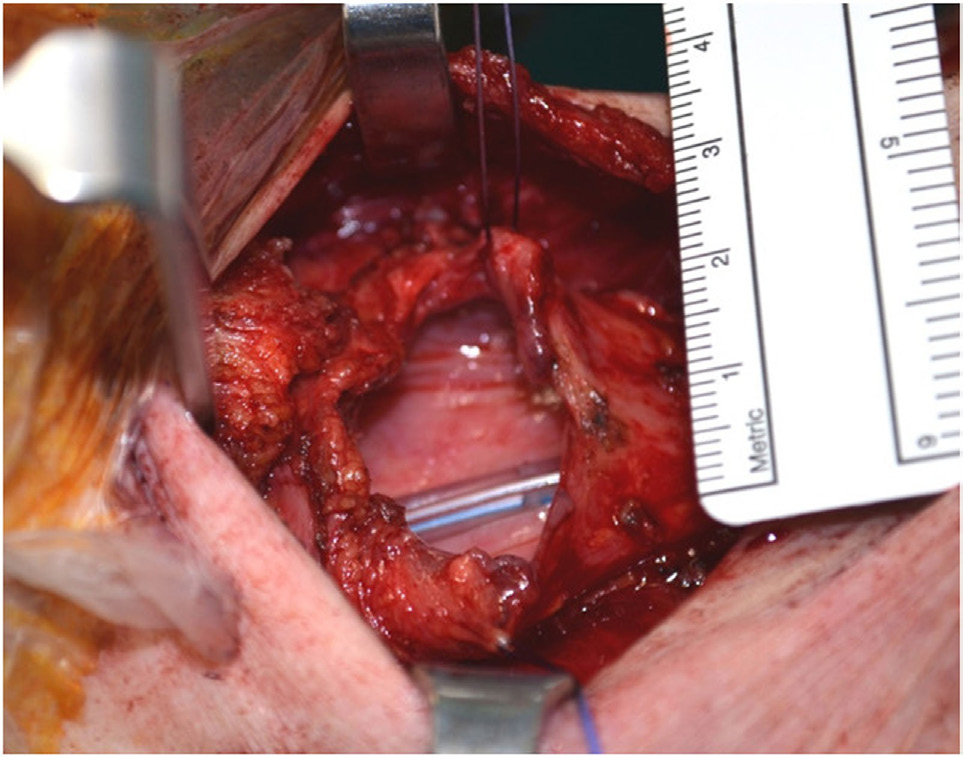
Small pharyngeal opening, about 5 cm that will be closed horizontally.

A tracheal esophageal puncture is performed, and the prosthesis is placed primarily. The pharyngotomy is closed horizontally, without any undue tension (Figure 6). The prelaryngeal muscles are sutured at the midline, and the tracheostomy and neck incisions closed.

### Study Population

We retrospectively analyzed all consecutive patients who underwent maximal mucosal-sparing total laryngectomy (MMSTL) between 2014 and 2016.

Demographic variables, previous treatments, and their complications, the reason for the functional laryngectomy, and possible complications were extracted from the patient’s records and tabulated (Table 1).

**Table 1:** Patient characteristics.

Age	Sex	Year of diagnosis	Carcinoma location	Stage	Carcinoma therapy	Comorbidities	Tracheostomy	Feeding tube	Reason for MMFTL	Fistula
81	M	1997 2008	Oral cavity Larynx	T4aN1 T1aN0	Resection + RT Laser cordectomy	Cachexia	No	Yes	Aspiration pneumonia	No
54	M	2010 2013	Oropharynx Recurrence	T2N1	RT Parotidectomy + radical neck dissection + carotid artery graft + ChemoRT	X + XII palsy	Yes	Yes	Aspiration pneumonia	No
69	M	2009 2014	Oropharynx Supraglottis	T2N2a T3N0	Neck dissection + ChemoRT Supraglottic laryngectomy	Lung cancer	Yes	Yes	Aspiration pneumonia	No
66	M	1997	Larynx	T2N0	ChemoRT	Stroke	Yes	Yes	Aspiration pneumonia	No
72	F	ALS with major swallowing disability RT 7.5 Gy to salivary glands				–	No	Yes	Aspiration pneumonia No speech + no oral feeding	No
70	F	2002 2003	Oropharynx Recurrence	T4aN2b	ChemoRT Neck dissection + carotid artery graft	Bilateral cord palsy Cachexia	No	Yes	Aspiration pneumonia	No
78	M	2015	Larynx	T3N0	ChemoRT	Parkinson Chondronecrosis	Yes	Yes	No speech + no oral feeding	Yes
81	M	2009 2014	Oropharynx Oral cavity Recurrence	T2N0 T2N0	ChemoRT Transoral resection Composite resection	–	No	Yes	No oral feeding + oropharyngeal stenosis and invalidating crusting	No
80	F	1998	Oropharynx	T1N2b	RT	Cachexia	No	No	Recurrent pharyngeal and supraglottic narrowing	No
59	F	2015	Larynx	T3N1	CHEP + ChemoRT	–	Yes	Yes	Aspiration pneumonia	No

## Results

Ten patients underwent MMSTL. Their average age was 71 ± 9 years. The average BMI was 18.7 ± 8. They all had a dysfunctional larynx and swallowing, with 90% already having a feeding tube and 50% having a tracheostomy in place.

The average duration of surgery was 121 ± 32 min. The average hospital stay was 20 ± 4 days for the entire cohort and 17 ± 2 days for the patients without fistula. All patients resumed oral diet and had their feeding tube removed.

Only one patient developed a postoperative fistula requiring a suprascapular pediculated fascio-cutaneous flap for closure.

## Discussion

Variables rates (3–66%) of pharyngocutaneous fistula after total laryngectomy have been reported (2), the average being about 14.3% (95% CI 11.7–17.0) (3). One meta-analysis concluded that postoperative hemoglobin level, prior tracheotomy, preoperative radiotherapy, and concurrent neck dissection were associated with increased fistula rates (2). Considering salvage total laryngectomy, the fistula rates vary between 14 and 61% (3), and a meta-analysis concluded to average fistula incidence of 27.6% (23.4–31.8) (3). Fistula rates after radiotherapy alone were 22.8% (18.3–27.4) and 34.1% (22.6–45.6) after chemoradiotherapy (3). Flap-reinforced closure decreased the fistula incidence to 10.3% (4.6–15.9) (3) and has become routine practice in the majority of centers.

Robotics-associated advantages aside, transoral total laryngectomy introduced the concept of mucosa sparing, which results in a minimal pharyngotomy defect and a horizontal closure, as well as minimal lateral dissection toward the neck and preservation of prelaryngeal muscles that in turn allow to minimize the risk of fistula. In the same year as the initial technical article, two cases of cancer-free dysfunctional larynx operated with a similar technique were published (4) as well as five cases, four of which had a recurrent carcinoma after chemoradiation (5). One of the cases described developed a postoperative fistula.

In our opinion, the surgical technique of open MMSTL outlined here retains all of the advantages of transoral robotic total laryngectomy: (1) minimal neck incisions; (2) maximal mucosa sparing; (3) minimal pharyngotomy defect; (4) minimal lateral dissection toward the carotid sheath; (5) horizontal closure; and (6) preservation of prelaryngeal muscles, allowing minimizing the risk of fistula.

We fail to grasp what advantages doing part of the surgery with an endoscopic exposure might accomplish. Having a slightly smaller (1–2 cm less?) neck incision and possibly a slightly smaller pharyngotomy defect is probably not of tremendous advantage. Furthermore, the amount of surgery done endoscopically vs. the amount of dissection through the neck opening is unclear in the published reports.

The major advantage of both approaches is to obliviate the need of flaps without increasing the rate of post-laryngectomy fistula. In the published literature, so far one of seven patients operated with transoral laryngectomy developed a fistula, an incidence comparable to our series, as well as to the cited 10% fistula rate after salvage total laryngectomy with flap coverage (3).

Other obvious advantages of open MMSTL are (1) quickness, with a procedure duration of about 2 h; (2) lack of endoscopic exposure problems, the previous radiation, and/or surgery precluding sufficient neck extension in majority of the targeted population; (3) no need of special training since the procedure steps are familiar to head and neck surgeons; and (4) equipment related costs.

We have used MMSTL only for functional laryngectomy and not for oncologic total laryngectomy. It is probably possible to modify the technique in some cases of salvage or primary laryngectomy for cancer, when the disease bulk is limited and does extend either anteriorly through the thyroid or cricoid cartilages or posteriorly to the arytenoids and retrocricoid region.

## Conclusion

Open maximal mucosal-sparing total laryngectomy without a flap is associated with low rates of postoperative fistula and seems to present several advantages to transoral robotic total laryngectomy.

## Ethics Statement

Retrospective chart studies of patients are waived of formal approval by the hospital ethics committee.

## Author Contributions

All the authors: conception of the work, analysis, and interpretation of data; final approval of the version to be published; agreement to be accountable for all aspects of the work in ensuring that questions related to the accuracy or integrity of any part of the work are appropriately investigated and resolved.

## Conflict of Interest Statement

The authors declare that the research was conducted in the absence of any commercial or financial relationships that could be construed as a potential conflict of interest.
